# Development and Validation of Nine-RNA Binding Protein Signature Predicting Overall Survival for Kidney Renal Clear Cell Carcinoma

**DOI:** 10.3389/fgene.2020.568192

**Published:** 2020-10-02

**Authors:** Weimin Zhong, Chaoqun Huang, Jianqiong Lin, Maoshu Zhu, Hongbin Zhong, Ming-Hsien Chiang, Huei-Shien Chiang, Mei-Sau Hui, Yao Lin, Jiyi Huang

**Affiliations:** ^1^The Fifth Hospital of Xiamen, Xiamen, China; ^2^Taiwan LinkMed Asia Public Health & Healthcare Management Research Association, Taipei, Taiwan; ^3^Far Eastern Polyclinic, Zhongzheng, Taiwan; ^4^Key Laboratory of Optoelectronic Science and Technology for Medicine of Ministry of Education, College of Life Sciences, Fujian Normal University, Fuzhou, China; ^5^Xiang’an Branch, The First Affiliated Hospital of Xiamen University, Xiamen University, Xiamen, China

**Keywords:** kidney renal clear cell carcinoma, differentially expressed RBP, protein-protein interaction network, survival analysis, nomogram, drugs

## Abstract

Cumulative studies have shown that RNA binding proteins (RBPs) play an important role in numerous malignant tumors and are related to the occurrence and progression of tumors. However, the role of RBPs in kidney renal clear cell carcinoma (KIRC) is not fully understood. In this study, we first downloaded gene expression data and corresponding clinical information of KIRC from the Cancer Genome Atlas (TCGA) database, International Cancer Genome Consortium (ICGC), and Gene Expression Omnibus (GEO) database, respectively. A total of 137 differentially expressed RBPs (DERBPs) were then identified between normal and tumor tissue, including 38 downregulated and 99 upregulated RBPs. Nine RBPs (EIF4A1, RPL36A, EXOSC5, RPL28, RPL13, RPS19, RPS2, EEF1A2, and OASL) were served as prognostic genes and exploited to construct a prognostic model through survival analysis. Kaplan-Meier curves analysis showed that the low-risk group had a better survival outcome when compared with the high-risk group. The area under the curve (AUC) value of the prognostic model was 0.713 in the TCGA data set (training data set), 0.706 in the ICGC data set, and 0.687 in the GSE29609 data set, respectively, confirming a good prognostic model. The prognostic model was also identified as an independent prognostic factor for KIRC survival by performing cox regression analysis. In addition, we also built a nomogram relying on age and the prognostic model and internal validation in the TCGA data set. The clinical benefit of the prognostic model was revealed by decision curve analysis (DCA). Gene set enrichment analysis revealed several crucial pathways (ERBB signaling pathway, pathways in cancer, MTOR signaling pathway, WNT signaling pathway, and TGF BETA signaling pathway) that may explain the underlying mechanisms of KIRC. Furthermore, potential drugs for KIRC treatment were predicted by the Connectivity Map (Cmap) database based on DERBPs, including several important drugs, such as depudecin and vorinostat, that could reverse KIRC gene expression, which may provide reference for the treatment of KIRC. In summary, we developed and validated a robust nine-RBP signature for KIRC prognosis prediction. A nomogram with risk score and age can be applied to promote the individualized prediction of overall survival in patients with KIRC. Moreover, the two drugs depudecin and vorinostat may contribute to KIRC treatment.

## Introduction

Renal cell carcinoma (RCC) is one of the most common cancers in people and mainly classified as three types: kidney renal clear cell carcinoma (KIRC), kidney renal papillary cell carcinoma (KIRP), and malignancies of the chromophobe. It has been reported that about 14,240 people died and 62,700 newly validated patients with kidney cancer were discovered in the United States in 2016 (Siegel et al., 2015). According to morphology, RCC can be mainly divided into three subtypes: KIRC, KIRP, and malignancies of the chromophobe (Fernandez-Pello et al., 2017; Foshat and Eyzaguirre, 2017). Among them, KIRC accounts for about 70%–80% kidney carcinoma. Moreover, KIRC patients have no obvious symptoms in the early stage, and about 30% of KIRC cases show metastasis when it is detected because of the sophisticated KIRC tumorigenesis in advanced stages (Ezz El Din, 2016; Zhao et al., 2016). Although some well-known biomarkers of KIRC, such as VHL/HIF, PI3K/Akt/mTOR, and Ras/Raf/MEK/ERK, have been identified, the underlying molecular mechanism of KIRC still remains uncertain (Elfiky et al., 2011; Colbert et al., 2015). Regarding the KIRC treatment, PD-1/PD-L1 blocking agents have been approved in the treatment of KIRC and in inhibiting the immune checkpoint have achieved some progress (Hahn et al., 2020). However, some patients still respond poorly, showing resistance to progress (Stein et al., 2020). Thus, it is necessary to reveal the underlying mechanism of KIRC to develop effective drugs or methods for its diagnosis and treatment.

RNA binding proteins (RBPs) are a class of proteins that interact with multiple types of RNAs. At present, it is reported that nearly 1500 RBPs were identified in the human genome (Gerstberger et al., 2014). The RBPs play a crucial role in preserving the physiological balance of cells, especially in the process of cell development and stress response (Masuda and Kuwano, 2019). Given the importance of post-transcriptional regulation, abnormal RBPs could lead to numerous human diseases. A previous study reveals that aberrant RBPs are associated with the occurrence and development of disease or cancers. For example, SRF1 and HuR can mediate post-transcriptional events to control the occurrence and progression of cardiovascular diseases (de Bruin et al., 2017). HuR can control mRNA stability to boost proliferation and metastasis of gastric cancer (Xie et al., 2019).

Currently, the potential role of RBP in KIRC is not fully understood, and a comprehensive functional study of RBP will help us fully understand its importance in the occurrence and development of KIRC. Thus, we firstly downloaded RNA sequencing data and the corresponding clinical information of KIRC from the TCGA, GEO, and ICGA databases. We then identified disregulated RBPs between normal and tumor tissue and systematically explored their prognostic values and molecular mechanisms in KIRC. Our study validated several prognostic RBPs that elevate our knowledge of the molecular mechanisms underlying KIRC.

## Materials and Methods

### Data Processing

We downloaded the read count of KIRC, including 72 normal and 539 tumor tissues with its corresponding clinical information from TCGA^1^ (Table 1). In order to identify DERBPs, we employed the edgeR R package to perform analyses (Robinson et al., 2010). The DERBPs were screened with the cutoff: | log fold change (FC)| ≥ 1 and false discovery rate (FDR) < 0.05. Moreover, we also downloaded 91 KIRC samples as a validation data set from the ICGC^2^.

**TABLE 1:** Statistics of clinical information in high risk group and low risk group.

Characteristic		High risk (*N* = 256)	Low risk (*N* = 256)	Total (*N* = 512)	*P* value
				
Age	<65	153	173	326	0.08082557
	> = 65	103	83	186	
Stage	Stage I	96	160	256	3.232756e-09
	Stage II	26	30	56	
	Stage III	73	45	118	
	Stage IV	61	21	82	
T	T1	99	161	260	4.090107e-08
	T2	35	33	68	
	T3	112	61	173	
	T4	10	1	11	
M	M0	184	222	406	4.780686e-06
	M1	58	20	78	
	MX	14	14	28	
N	N0	119	109	228	0.3058459
	N1	11	5	16	
	NX	126	142	268	
Gender	Female	83	93	176	0.4023477
	Male	173	163	336	
Grade	G1	2	9	11	2.814139e-06
	G2	91	128	219	
	G3	109	94	203	
	G4	54	19	73	
	GX	0	6	6	
Smoking	1-year	135	130	265	0.8603639
	2-year	11	14	25	
	3-year	91	92	183	
	4-year	14	12	26	
	5-year	5	8	13	

### KEGG Pathway, GO Enrichment Analysis, GSEA Enrichment, and PPI Network Construction

The potential function of the DERBPs was further applied to GO enrichment and Kyoto Encyclopedia of Genes and Genomes (KEGG) pathway analysis using clusterProfiler R package (Yu et al., 2012). Both *p* and FDR values less than 0.05 were statistically significant. To further screen the key module for RBPs, the DERBPs were uploaded to the STRING database^3^ first (Szklarczyk et al., 2019). The Cytoscape software was further employed to build a ppi network (Smoot et al., 2011). The crucial modules were screened by using the Molecular Complex Detection (MCODE) module with the criterion score ≥ 2. GSEA enrichment analysis was performed among different risk groups, and a significant pathway was selected with the NOM-*p* value < 0.05 and FDR < 0.05.

### Survival Analysis

By integrating clinical information and RBP expression profiles, we first performed univariate cox regression analysis using the survival R package and selected those significant RBPs with its *p* value smaller than or equal to 0.05. Then, in order to increase the feasibility and reliability of the clinical prognosis based on RBPs, we conducted a robust likelihood-based survival analysis to further selected target RBPs by using the Rbsurv R package (Renaud et al., 2015). The procedure was as follows:

1. All the samples were randomly categorized into the training set with N^∗^(1-p) samples and a testing data set with N^∗^p samples. We fitted a gene into the training data set and obtained its parameter estimation. Subsequently, we estimated the log likelihood with the parameter estimate and the validation set of samples. This evaluation was repeated 10 times for each gene, and we obtained 10 log likelihoods for each gene.
2. The best gene, g (Siegel et al., 2015), with the corresponding largest mean log likelihood was selected. We then searched the next best gene by evaluating every two-gene model and selected an optimal one with the largest mean log likelihood. A series of predictive models was built based on the above procedure, and the Akaike information criterion (AIC) value for each gene was calculated. The optimal model was screened with the lowest AIC value. Using this model, the prognostic RBPs were strictly selected.

After selecting most predictive genes, Multivariate cox regression analysis was conducted on these RBPs to calculate the corresponding coefficient. According to the coefficient, we constructed the risk score system and the formula as follows: Risk score = ΣCoef_RBPs_ x Exp_RBPs._ In the risk score formula, the Coef_RBPs_ represent the regression coefficients of each RBP, and Exp_RBPs_ is the expression level of each prognostic RBP. Subsequently, we calculated the risk score for each patient and further categorized the patient into a high- or low-risk score group based on the median score. In addition, we performed an ROC curve analysis by using the survivalROC R package to estimate the sensitivity and specificity of the prognostic RBP risk model^4^. Log-rank p < 0.05 was considered significant for survival analysis.

### Independence of the Risk Model of Other Clinical Parameters in TCGA

In order to evaluate the independence of the risk model, we compared clinical features, such as age, gender, grade, and stage with the risk model using the univariate and multivariate cox regression analyses, and *p* < 0.05 were considered statistically significant.

### Building and Validating a Predictive Nomogram

A nomogram was built by including all significantly independent prognostic factors (Iasonos et al., 2008). The calibration plot was applied to explore the calibration and the discrimination of the nomogram. The age, prognostic, and combined models (age and prognostic model) were compared with ROC and decision curve analyses (DCA) (Vickers and Elkin, 2006).

### External Validation of the Prognostic Gene Signature

The validation data sets were downloaded from ICGC with 91 samples and GSE29609 with 39 patient samples. We then calculated the risk score for each patient based on the prognostic model. Then the ROC and Kaplan-Meier analyses, respectively, were performed in the ICGA data set. In addition, the protein expression of the prognostic genes in the risk model was further validated in the Human Protein Atlas (HPA, https://www.proteinatlas.org/) (Nwosu et al., 2017). The online tool cBioportal was used to explore the genetic alterations of the prognostic genes^5^.

### Identification of Candidate Small Molecules

The CMap database^6^ was applied to predict a potential drug that may reverse or induce the biological states of KIRC based on the gene expression (Lamb et al., 2006). We first uploaded the DERBPs to the CMAP in the “query” module and then searched for small molecular drugs that may treat KIRC. The enrich scores ranging from -1 to 1 represent the correlation level between drugs and DERBPs. Drugs with a greater negative correlation value are more beneficial for the treatment of KIRC. Therefore, drugs with a score of ≤-0.75 were considered as candidate drugs for KIRC treatment. In addition, we also performed mode-of-action (MoA) analysis for the drugs to search for the potential mechanism.

## Results

### Identification of DERBPs in KIRC

**FIGURE 1:**
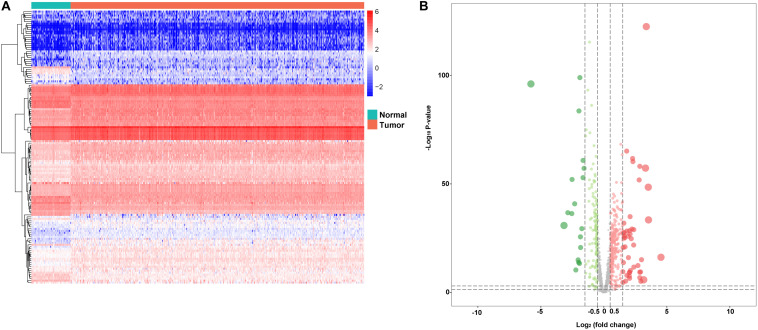
Differentially expressed RBPs. **A** Heat maps of differentially expressed RBPs between tumor and normal tissues in the TCGA data set. **B** Volcano plot of differentially expressed RBPs; red dots represent upregulated RBPs, and green dots represent downregulated RBPs.

In the study, we collected 72 normal tissues and 539 tumors of KIRC from TCGA database. To explore the DERBPs, we compared the RBP gene expression between normal and tumor tissue using the edegR R package. As a result, a total of 137 DERBPs were obtained with the cutoff: | logFC| > 1 and FDR < 0.05, of which 38 RBPs were downregulated and 99 were upregulated. The expression distribution of these differently expressed RBPs is shown in Figures 1A,B.

### GO and KEGG Enrichment for DERBPs

In order to explore the potential function of the DERBPs, we use the clusterProfiler R package to perform functional enrichment analysis. As a result, these RBPs were mainly enriched in translational initiation, mRNA catabolic processes, RNA catabolic processes, nuclear-transcribed mRNA catabolic processes, SRP-dependent co-translational protein targeting to membrane, and co-translational protein targeting to membrane (Supplementary Figure 1A). Moreover, we also discovered that these RBPs were involved in ribosome and legionellosis pathways in the KEGG result, which is consistent with the previous study (Supplementary Figure 1B).

### Construction Protein–Protein Interaction (PPI) Network and Crucial Modules Screening

**FIGURE 2:**
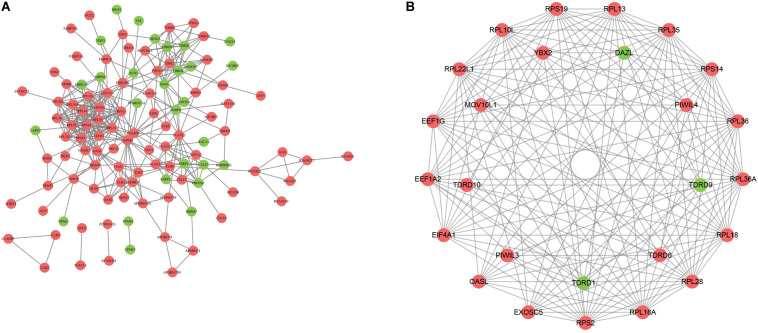
Construction of protein–protein interaction network. **A** The network visualization using cytoscape for all differentially expressed RBPs. **B** The network of two key modules visualized by cytoscape.

To explore the role of DERBPs, we uploaded the RBPs to the String database and identified a PPI network. We further used the Cytoscape software to visualize it (Figure 2A). For the purpose of searching the key modules from the PPI network, we used the MCODE module to identify the important modules. As a result, the top two important modules were acquired, which consist of 26 potential key RBPs (Figure 2B).

### Identification and Selection of Prognostic Related RBPs

In order to obtain a reliable survival result for KIRC, we first excluded samples with a survival time less than 30 days. As a result, a set of 26 RBPs with 512 patients were exploited into univariate cox regression analysis, and a total of 10 significant RBPs were identified (*p* < 0.05) (Supplementary Table 1). To ensure the stability and feasibility of clinical prognosis based on 10 RBPs, we further made a selection on the 10 RBPs using the robust likelihood-based survival analysis. As shown in Table 2, nine genes, including EIF4A1, RPL36A, EXOSC5, RPL28, RPL13, RPS19, RPS2, EEF1A2, and OASL, were picked out. To systemically investigate the relationship between these nine RBPs and prognosis of KIRC, we developed a nine-RBP signature-based risk score based on their cox coefficient:

**TABLE 2:** Prognostic RBPs signature screened by performing forward selection analysis in the TCGA dataset.

Gene ID	nloglik	AIC
EIF4A1	943.86	1889.73*
RPL36A	935.21	1874.43*
EXOSC5	934.78	1875.56*
RPL28	933.65	1875.3*
RPL13	933.65	1877.3*
RPS19	933.58	1879.15*
RPS2	932.74	1879.49*
EEF1A2	929.78	1875.55*
OASL	926.6	1871.2*

Risk score = (0.005121079 ^∗^ EIF4A1)

+ (−0.065342266 ^∗^ RPL36A)

+ (−0.074842527 ^∗^ EXOSC5)

+ (−0.007007688 ^∗^ RPL28)

+ (0.003365894 ^∗^ RPL13)

+ (−0.000184204 ^∗^ RPS19)

+ (0.000510318 ^∗^ RPS2)

+ (0.017008893 ^∗^EEF1A2)

+ (0.118627759 ^∗^ OASL)

**FIGURE 3:**
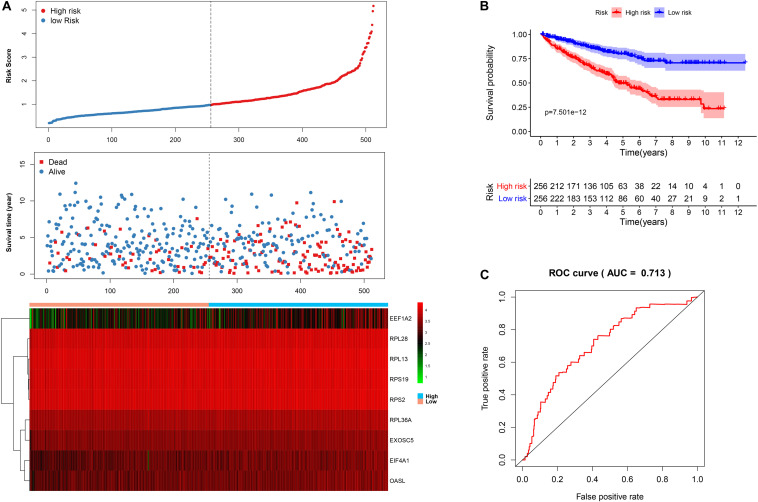
The nine-RBP signature associated with overall survival of KIRC in the TCGA data set. **A** The upper panel represents the risk score distribution for each patient, the middle panel shows the patient distribution with increasing risk value, and the lower panel represents the expression of nine prognostic RBPs. **B** Kaplan-Meier curve analysis for the patients in KIRC between the high- and low-risk groups. **C** ROC curve analysis for the prognostic model.

**FIGURE 4:**
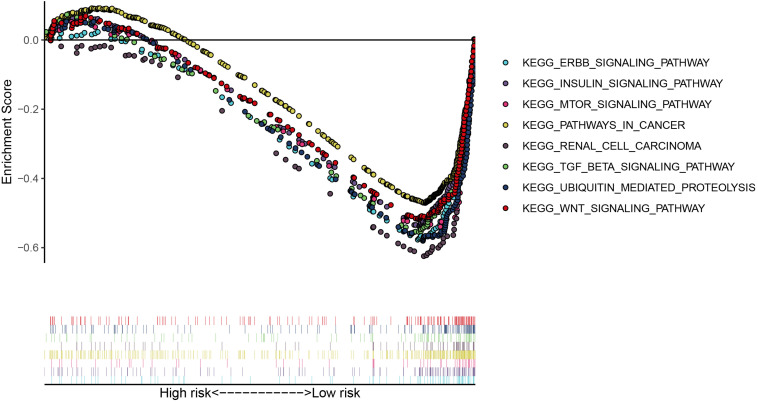
The significant pathways were enriched in the low-risk group by performing the GSEA analysis based on the gene expression.

**FIGURE 5:**
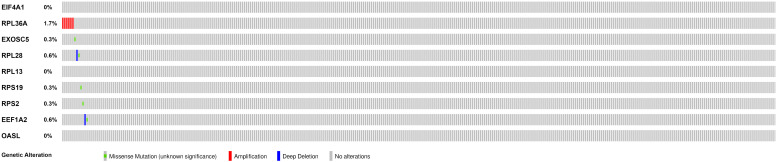
Genetic alterations of the nine RBPs in KIRC patients; the data were retrieved from the cBioportal database.

We then calculated the risk score for each patient based on the risk formula and ranked them according to the risk score. Figure 3A shows that survival time of patients with KIRC was affected adversely with their risk score. Numerous cases of death were related to a high-risk score, and patients with a low-risk score have prolonged survival time. The Kaplan-Meier curve and log-rank test indicated that patients in the low-risk group have a better survival time than in the high-risk group (*p* < 0.01) (Figure 3B). To compare the sensitivity and specificity of survival prediction, ROC analysis was performed for the nine-RBP signature. As shown in Figure 3C, the area under the curve (AUC) values reached 0.713, exhibiting a good accuracy. In addition, to further explore the function between the high- and low-risk group, we performed GSEA enrichment and found several important pathways, including the insulin signaling pathway, ERBB signaling pathway, renal cell carcinoma, pathways in cancer, MTOR signaling pathway, WNT signaling pathway, TGF BETA signaling pathway, and UBIQUITIN mediated proteolysis that were enriched in the low-risk group (Figure 4). We then assessed the alterations in nine genes by using the cBioPortal database as shown in Figure 5, and the RPL36A gene included six amplification samples; RPL28 and EEF1A2 were altered in 0.6% of cases, and EXOSC5, RPS19, and RPS2 were altered in 0.3% cases while EIF4A1 and OASL have no mutation cases.

### Independent Prognostic Role of the Prognostic RBP Signature

**FIGURE 6:**
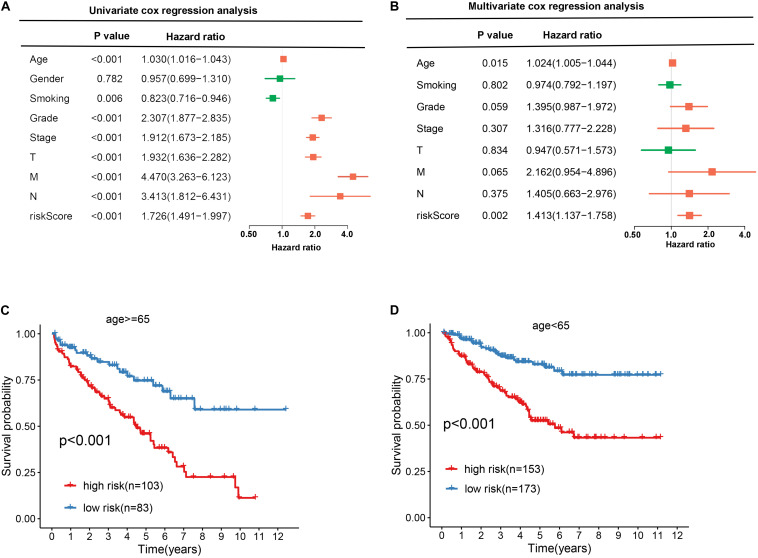
Exploring the association between clinical traits and the prognostic model. **A** Univariate cox regression analysis between clinical traits and prognostic risk score. **B** Multivariate cox regression analysis between clinical traits and prognostic risk score. **C, D** The Kaplan–Meier curve shows the prognostic value of the nine-RBP signature for KIRC patients categorized by age.

To explore the independence of the signature, we compared the clinical features including gender, age, smoking, grade, stage, T, N, M, and RBP signature by performing univariate and multivariate cox regression analysis. As shown in Figures 6A,B, the age and RBP signature risk score were considered as the independent prognostic factor (*p* < 0.05). Then patients were stratified according to age (<65 and ≥65). Patients in the high-risk group shown significantly poorer OS than those in the low-risk group both in <65 and in ≥65 (Figures 6C,D).

### Construction of a Nomogram Based on Prognostic Model and Clinical Features

**FIGURE 7:**
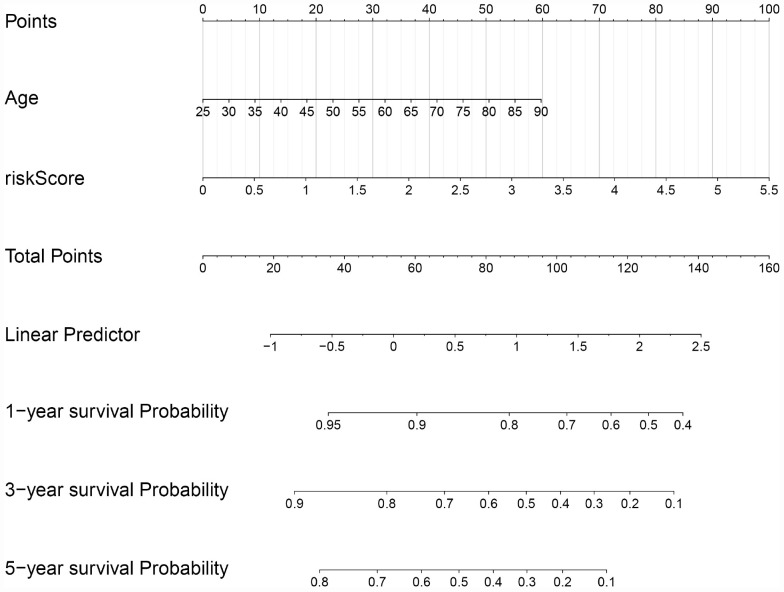
A nomogram plot was constructed on the basis of two independent prognostic factors (age and prognostic risk score) in KIRC.

**FIGURE 8:**
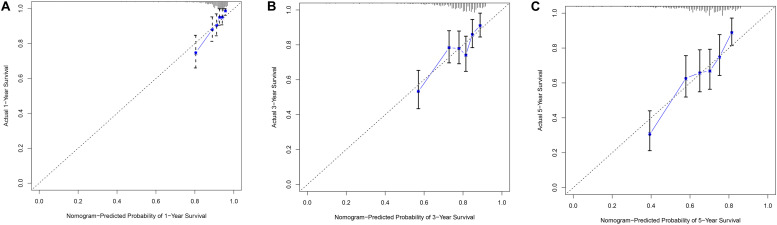
The calibration plot for internal validation of the nomogram within 1-year **(A)**, 3-year **(B)** and 5-year **(C)**, respectively.

**FIGURE 9:**
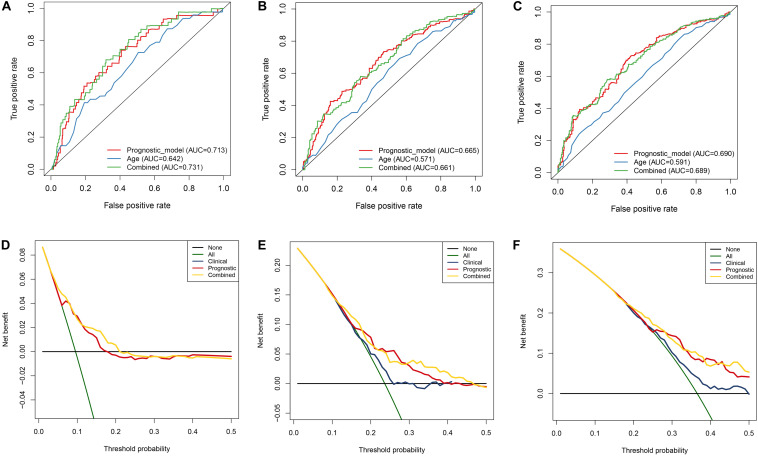
Estimation of nomogram by performing ROC curve and DCA curve analyses within 1, 3, and 5 years, respectively. **A–C** ROC curves analysis of the nomogram compared for 1, 3, and 5 years. **D–F** DCA curve analysis of the nomogram compared for 1, 3, and 5 years.

In order to evaluate the clinical trait and prognostic model for KIRC prognosis, we integrated the prognostic model and age to build a nomogram (Figure 7). In addition, the corresponding calibration plots in 1, 3, and 5 year were also drawn, and it was discovered that the performance of the nomogram was best in predicting 1 year OS (Figures 8A–C). We further estimate the AUC value for the age and prognostic model, respectively. As shown in Figures 9A–C, the AUC values for 1-, 3-, and 5-year OS were 0.64, 0.57, and 0.59 in age, and in the prognostic model, the AUC value for 1-, 3-, and 5-year OS were 0.71, 0.66, and 0.69, respectively. Interestingly, when we incorporated the age and prognostic model into a combined model, the AUC value in 1, 3, and 5 years was increased, especially in 1-year OS (Figures 9A–C). Moreover, we also discovered that combining our prognostic model with age showed some net benefit for predicting OS (Figures 9D–F).

### Validation of the Prognostic Model and Hub RBPs

**FIGURE 10:**
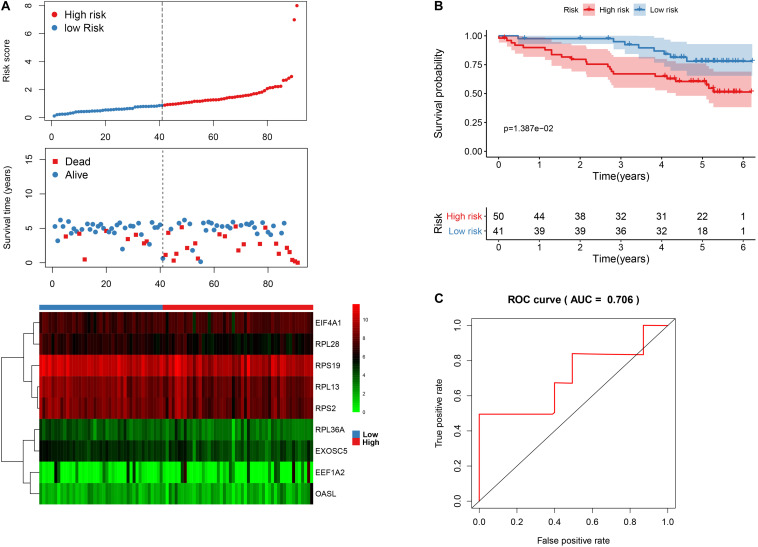
The nine-RBP signature associated with overall survival of KIRC in the ICGC data set. **A** The upper panel represents the risk score distribution for each patient, the middle panel shows the patients’ distribution with increasing risk value, and the lower panel represents the expression of nine prognostic RBPs. **B** Kaplan-Meier curves analysis for the patients in KIRC between the high- and low-risk groups. **C** ROC curve analysis for the prognostic model.

In order to validate the stability and reliability of the prognostic model, we first downloaded 91 samples with complete clinical information as the validation data set from the ICGC database. Using the prognostic model, we calculated the risk score for each patient and divided patients into high- and low-risk group, respectively. We found that patients in the high-risk group corresponded to higher death rates (Figure 10A). The Kaplan-Meier curve and log-rank test suggest that patients in the high-risk group have a worse survival rate compared to the low-risk group (*p* < 0.05) (Figure 10B). Moreover, the AUC for overall survival was reached in 0.706, indicating good accuracy (Figure 10C). Similarly, we also downloaded a GSE29609 data set from the GEO database that included 39 samples. According to the risk model, we also calculated the risk score for each patient and then classified into them high- and low-risk group, respectively. The Kaplan-Meier curve and log-rank test suggest that patients have a significant difference between risk groups (*P* < 0.05) (Supplementary Figure 2A). The ROC analysis results indicate good accuracy of the risk model for the prognosis of KIRC (Supplementary Figure 2B).

**FIGURE 11:**
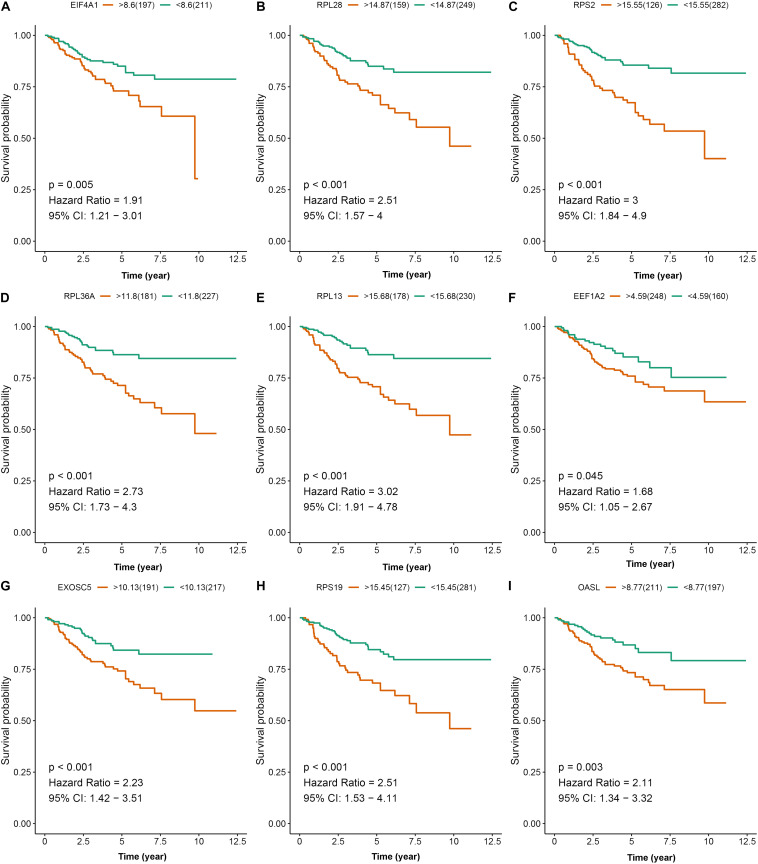
Disease-free survival analysis of the nine prognostic RBPs including EIF4A1 **(A)**, RPL28 **(B)**, RPS2 **(C)**, RPL36A **(D)**, RPL13 **(E)**, EEF1A2 **(F)**, EXOSC5 **(G)**, RPS19 **(H)**, and OASL **(I)** in the TCGA data set.

To further explore the prognostic value of nine hub RBPs in KIRC, we used the Kaplan-Meier curve and log-rank test analyses to determine the association between hub RBPs and disease-free survival (DFS). As shown in Figure 11, the nine hub RBPs were significantly associated with the DFS in KIRC patients, and high expression of them corresponded to a lower survival probability (*p* < 0.05). We also evaluated the expression level of the nine hub RBPs between tumor and normal tissue. As shown in Supplementary Figure 3, most of the hub RBPs presented significant divergence between normal and tumor tissue except for EEF1A2. Interestingly, these RBPs show a high expression level in tumor tissue when compared to normal tissue.

In addition, we further explore the protein expression of nine hub RBPs. We employed immunohistochemistry results from the HPA database to discover that EIF4A1 was significantly increased in kidney tumor tissue compared with normal tissue (Supplementary Figure 4). However, the antibody staining level of EEF1A2 and RPL36A were decreased from normal tissue to kidney tumor tissue (Supplementary Figure 4). Moreover, the protein expression level of EXOSC5, RPL13, RPL28, and RPS2 were not significant between normal and tumor tissue, and EXOSC5 and RPS19 were not detected in the HPA database.

### Related Drugs Screening for KIRC Treatment

**FIGURE 12:**
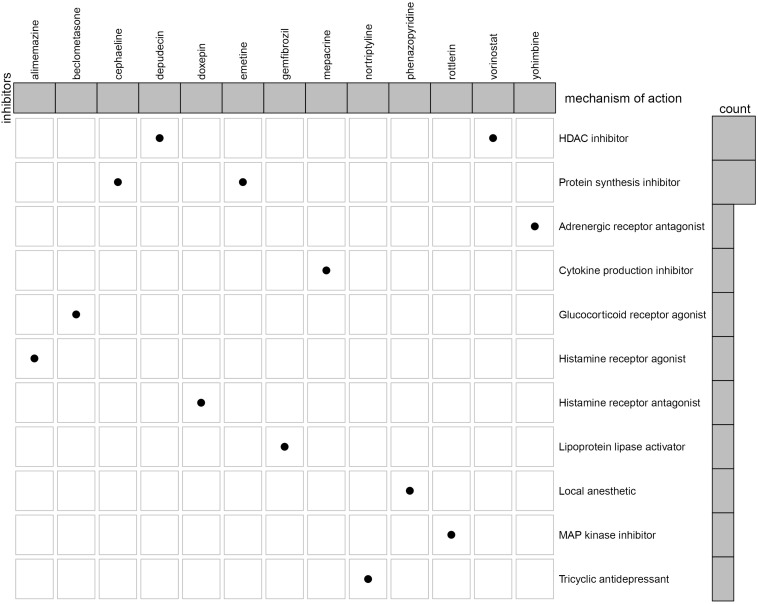
Oncoplot of the mechanism of action for the potential drugs.

To identify the potential drugs for KIRC, we uploaded the upregulated and downregulated RBPs to the CMAP database. As a result, 27 significant candidate drugs that score ≤ -0.50 and *p* value < 0.05 were considered as potential drugs for KIRC treatment (Supplementary Table 2). The mechanism of action for these drugs were further analyzed and are shown in Figure 12. We can discover that these drugs were enriched in the HDAC inhibitor, protein synthesis inhibitor, adrenergic receptor antagonist, cytokine production inhibitor, glucocorticoid receptor agonist, histamine receptor agonist, histamine receptor antagonist, lipoprotein lipase activator, local anesthetic, MAP kinase inhibitor, and Tricyclic antidepressant (Figure 12). These mechanisms of action and potential small molecule drugs might provide guidance for developing targeted drugs for KIRC.

## Discussion

Disorders of RBPs have been reported in numerous malignant tumors (Gerstberger et al., 2014; Masuda and Kuwano, 2019). However, fewer studies have comprehensively investigated the function and prognosis of RBPs. In the present study, we systemically explored the prognosis and function of hub RBPs in KIRC. A total of 137 DERBPs were identified between tumor and normal tissue of KIRC based on the TCGA RNAseq data. We comprehensively investigate the potential function and pathway and construct a PPI network for these RBPs. Furthermore, we constructed and validated a nine-RBP signature to predict KIRC prognosis based on the cox regression coefficient using the univariate cox regression analysis, robust likelihood-based survival analysis, multivariate cox regression analysis, and ROC analysis. We also identified some potential drugs that may contribute to treatment of KIRC. These findings might provide new insight into the pathogenesis of KIRC and potential therapeutic targets for KIRC.

Functional enrichment analysis results reveal that the DERBPs were mainly enriched in translation initiation, mRNA catabolic processes, RNA catabolic processes, nuclear-transcribed mRNA catabolic processes, SRP-dependent co-translational protein targeting to membrane, and cotranslational protein targeting to membrane, etc. Previous studies have demonstrated that regulation of translation, RNA processing, and the RNA metabolism process were the causes of the occurrence and development of the human disease (Jain et al., 2019; Siang et al., 2020). The KEGG analysis results indicate that the dysregulated RBPs were enriched in Ribosome and Legionellosis, which is consistent with previous studies (Li et al., 2020).

In addition, we constructed a PPI network for these DERBPs and identified two key modules with 26 hub RBPs. We further explored the association between 26 RBPs and overall survival of KIRC by performing univariate Cox regression analysis, robust likelihood-based survival analysis, and multivariate Cox regression analysis. A total of nine RBPs, including EIF4A1, RPL36A, EXOSC5, RPL28, RPL13, RPS19, RPS2, EEF1A2, and OASL, were identified as prognostic RBPs. Among the nine RBPs, EEF1A2, and RPL13 have been reported to be associated with tumorigenesis and progression of kidney cancer patients (Pflueger et al., 2013; Wierzbicki et al., 2014). Eukaryotic translation initiation factor 4A1 (EIF4A1) is a component of the translation initiation complex, and a high expression level of EIF4A1 is positively associated with poor tumor differentiation, late T stage, lymph node metastasis, advanced TNM stage, and poor prognosis in patients with gastric cancer (Gao et al., 2020). Overexpression of ribosomal protein L36a (RPL36A) has been reported to closely relate to cellular proliferation in hepatocellular carcinoma (Kim et al., 2004). The EXOSC5 was identified as a novel prognostic marker that can promote proliferation of colorectal cancer through activating the ERK and AKT pathways (Pan et al., 2019). The mutation of RPL28 was associated with shorter progression-free survival and overall survival in metastatic colorectal cancer (Labriet et al., 2019). Markiewski et al. found that the ribosomal protein S19 (RPS19) can contribute to generate regulatory T cells while reducing infiltration of CD8 + T cells into tumors. When the expression level of RPS19 is decreased, the tumor growth is impaired, and the development of tumors is also delayed in a transgenic model of breast cancer (Markiewski et al., 2017). The RPS2 and OASL were considered to be a potential therapeutic target in prostate cancer and lung cancer (Lv et al., 2018). According to the nine genes, we built a risk model with their coefficient. The ROC analysis results in the TCGA data set and ICGC data set revealed that our risk model has a good performance to predict survival of KIRC.

The GSEA result revealed many significant cancer-related pathways for the RBP signature, of which the insulin signaling pathway, ERBB signaling pathway, renal cell carcinoma, pathways in cancer, MTOR signaling pathway, WNT signaling pathway, TGF BETA signaling pathway, and UBIQUITIN mediated proteolysis were enriched in the low-risk group, and no significant pathway was enriched in the high-risk group. On one hand, these results demonstrate the robust connection of the RBP signature with tumorgenesis and progression of KIRC. On the other hand, the results might provide promising directions to elaborate the underlying molecular mechanisms of the signature.

To identify potential drugs for KIRC treatment, we obtained 27 compounds from the prediction of the CMAP database based on the DERBPs. Among these compounds, vorinostat, a histone deacetylase (HDAC) suppressor, has been reported to be a promising drug in the treatment of KIRP (Pang et al., 2019). The HDACs are a class of enzymes in the nucleus of eukaryotic organisms that promote histone deacetylation and, accordingly, allow histones to assemble and convert DNA into biologically active units (Valenzuela-Fernandez et al., 2008). According to the report, HDACs (HDAC1 and HDAC2) are necessary for the growth and survival of RCC tumor cells, and inhibition of HDACs might improve the response of oncologic chemotherapy for RCC (Aggarwal et al., 2017; Kiweler et al., 2018). Interestingly, depudecin is also an HDAC suppressor, which contributes to inducing morphological reversion of transformed fibroblasts and has been used to treat neuroendocrine tumor (Kwon et al., 1998; Kunnimalaiyaan and Chen, 2007). A recent study reports that depudecin can serve as a candidate drug for the treatment of pituitary adenomas (Zhou et al., 2016). The present study indicates a close reverse mechanistic association of depudecin and vorinostat with KIRC, suggesting that the two drugs may serve as suitable drugs for KIRC treatment. However, the mechanism and efficacy of the two drugs for treatment of KIRC remain to be elucidated in future studies.

Overall, we constructed an RBP prognostic model based on bioinformatics analysis, which have potentially substantial clinical significance. However, several limitations need to be pointed out. First, all the results were based on analysis, and further experimental verification is required. Second, the data sets did not provide complete clinical information, especially in the validation data set, which may reduce the statistical reliability and validity of the result.

## Conclusion

In conclusion, our study presents the expression, function, and prognostic potential of RBPs in KIRC. We identified a novel nine-RBP signature for KIRC and proved that the prognostic model can serve as an independent predictor for KIRC. To our knowledge, this is the first attempt to develope an RBP prognostic model in KIRC. In addition, we also identified two prospective drugs for the treatment of KIRC.

## Data Availability Statement

Publicly available datasets were analyzed in this study. This data can be found here: https://portal.gdc.cancer.gov, https://www.ncbi.nlm.nih.gov/geo/, and https://dcc.icgc.org/.

## Author Contributions

YL and JH designed the study. CH, JL, MZ, and HZ collected the clinical information and expression data. WZ analysis data and wrote the manuscript. M-HC, H-SC, and M-SH revised and offered advice about the manuscripts. All authors contributed to the article and approved the submitted version.

## Conflict of Interest

The authors declare that the research was conducted in the absence of any commercial or financial relationships that could be construed as a potential conflict of interest.

## Footnotes

1 https://portal.gdc.cancer.gov/

2 https://icgc.org/

3 http://www.string-db.org/

4 https://cran.rproject.org/web/packages/survivalROC/index.html

5 https://www.cbioportal.org/

6 http://www.broadinstitute.org

## Supplementary Material

Supplementary Figure 1 GO **(A)** and KEGG pathway **(B)** enrichment analysis for the differentially expressed RBPs.

Supplementary Figure 2 The nine RBP signature associated with overall survival of KIRC in the GSE29609 data set; **(A)** Kaplan-Meier curve analysis for the patients in KIRC between the high- and low-risk groups. **B** ROC curve analysis for the prognostic model.

Supplementary Figure 3 Exploration of the expression of the nine hub RBPs in normal and tumor tissue.

Supplementary Figure 4 Validation of the seven RBPs on the protein expression level based on Immunohistochemical results in KIRC; the data was retrieved from HPA database.

Supplementary Table 1 Univariate cox regression analysis for the hub RBPs.

Supplementary Table 2 Enrichment analysis for the 27 potential drugs in KIRC using the CMAP database.
