# Supplementary figures and images for: Development and Validation of Nine-RNA Binding Protein Signature Predicting Overall Survival for Kidney Renal Clear Cell Carcinoma
Source: Front Genet. 2020 Oct 2;11:568192. doi: 10.3389/fgene.2020.568192 (PMC7566920; doi:10.3389/fgene.2020.568192)

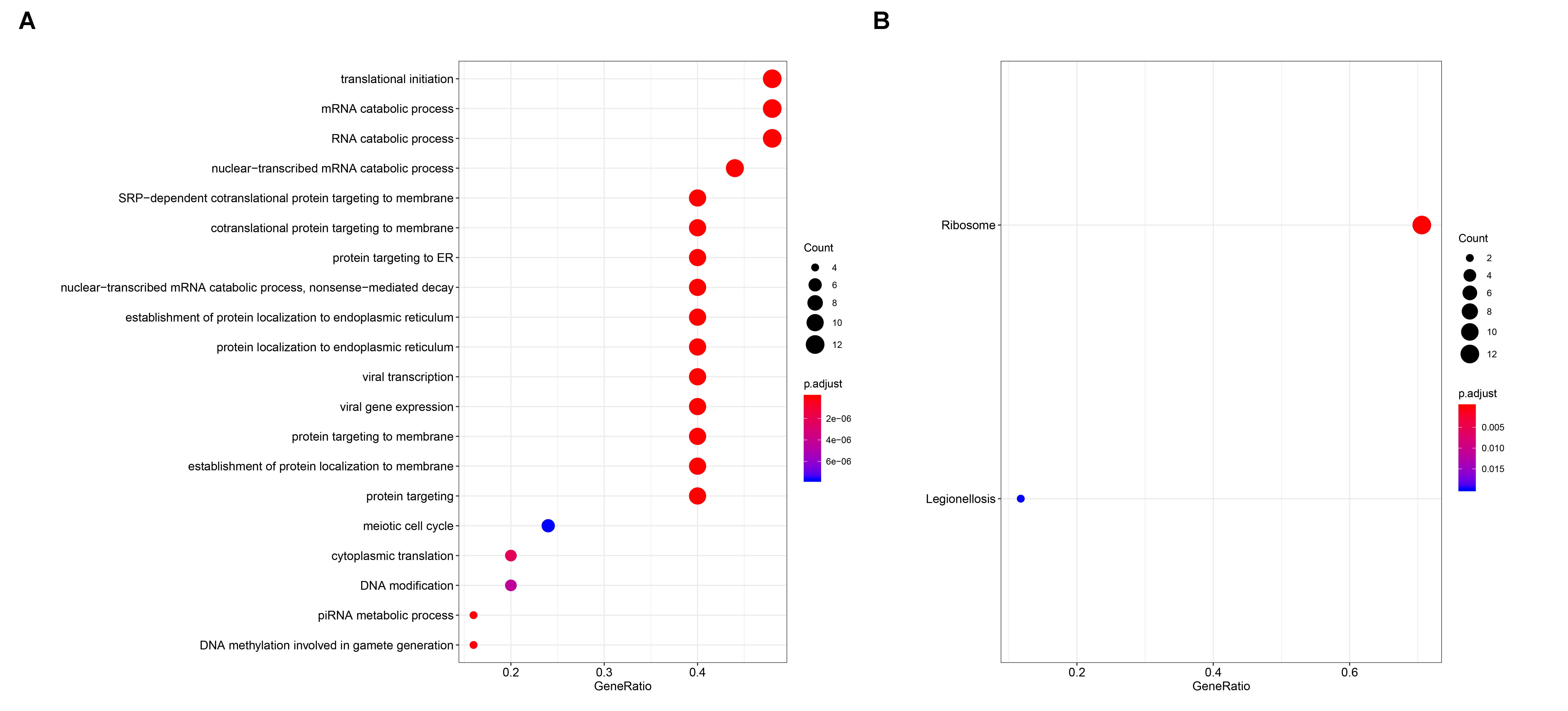

Supplement: Supplementary Figure 1 — GO (A) and KEGG pathway (B) enrichment analysis for the differentially expressed RBPs. [file Image_1.JPEG]

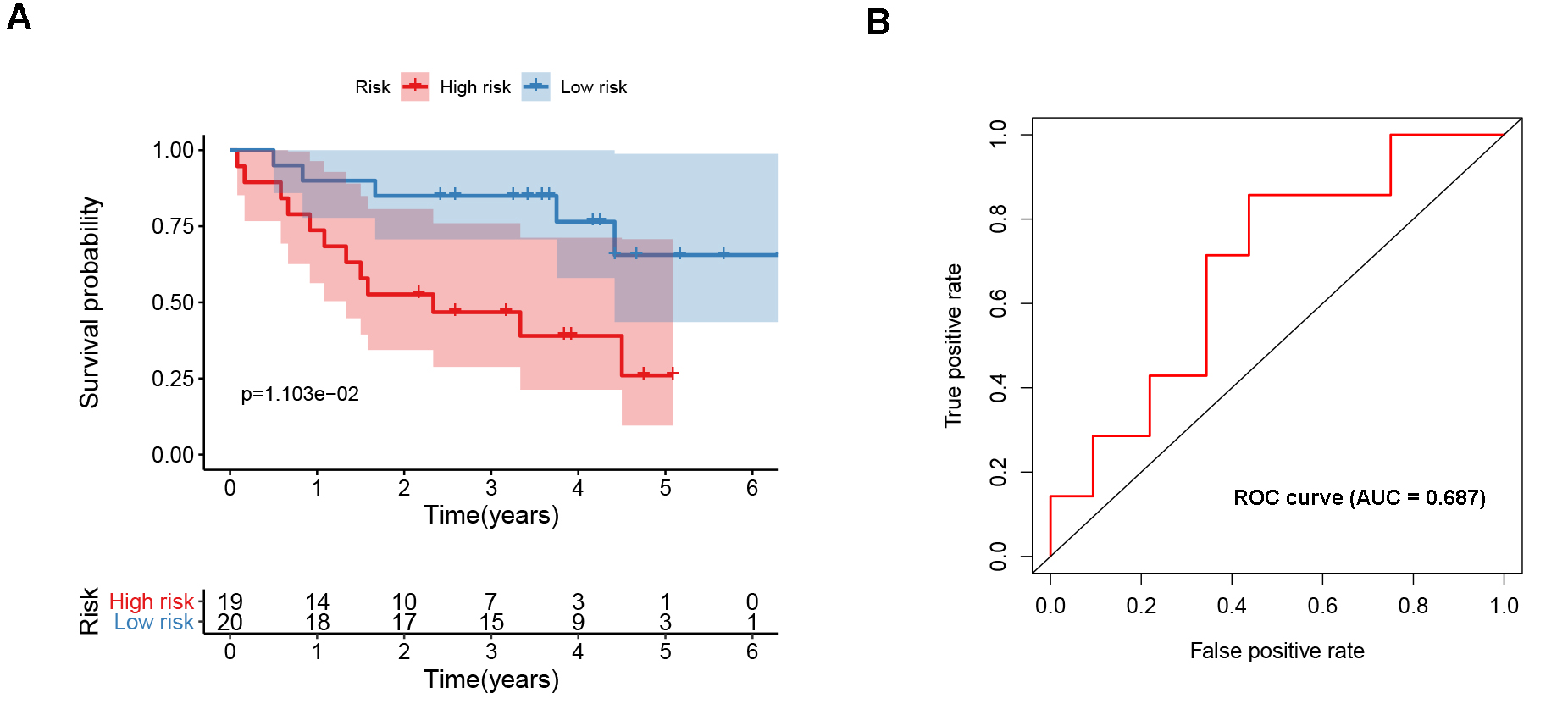

Supplement: Supplementary Figure 2 — The nine RBP signature associated with overall survival of KIRC in the GSE29609 data set; (A) Kaplan-Meier curve analysis for the patients in KIRC between the high- and low-risk groups. B ROC curve analysis for the prognostic model. [file Image_2.JPEG]

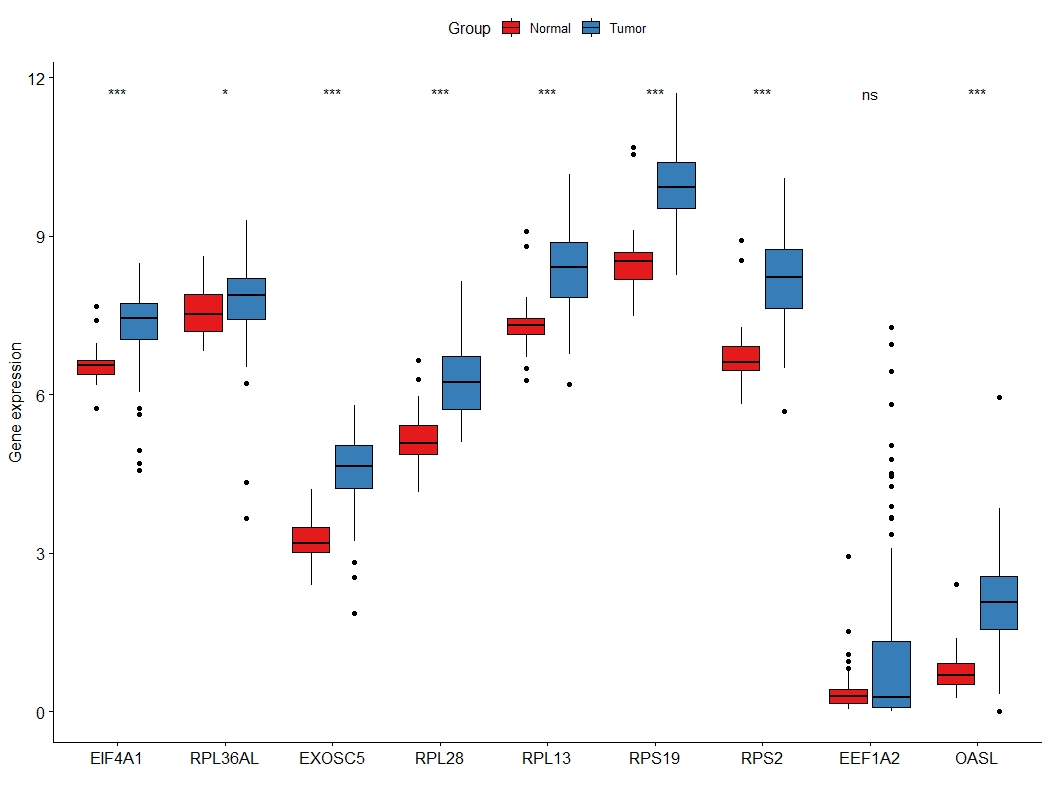

Supplement: Supplementary Figure 3 — Exploration of the expression of the nine hub RBPs in normal and tumor tissue. [file Image_3.JPEG]

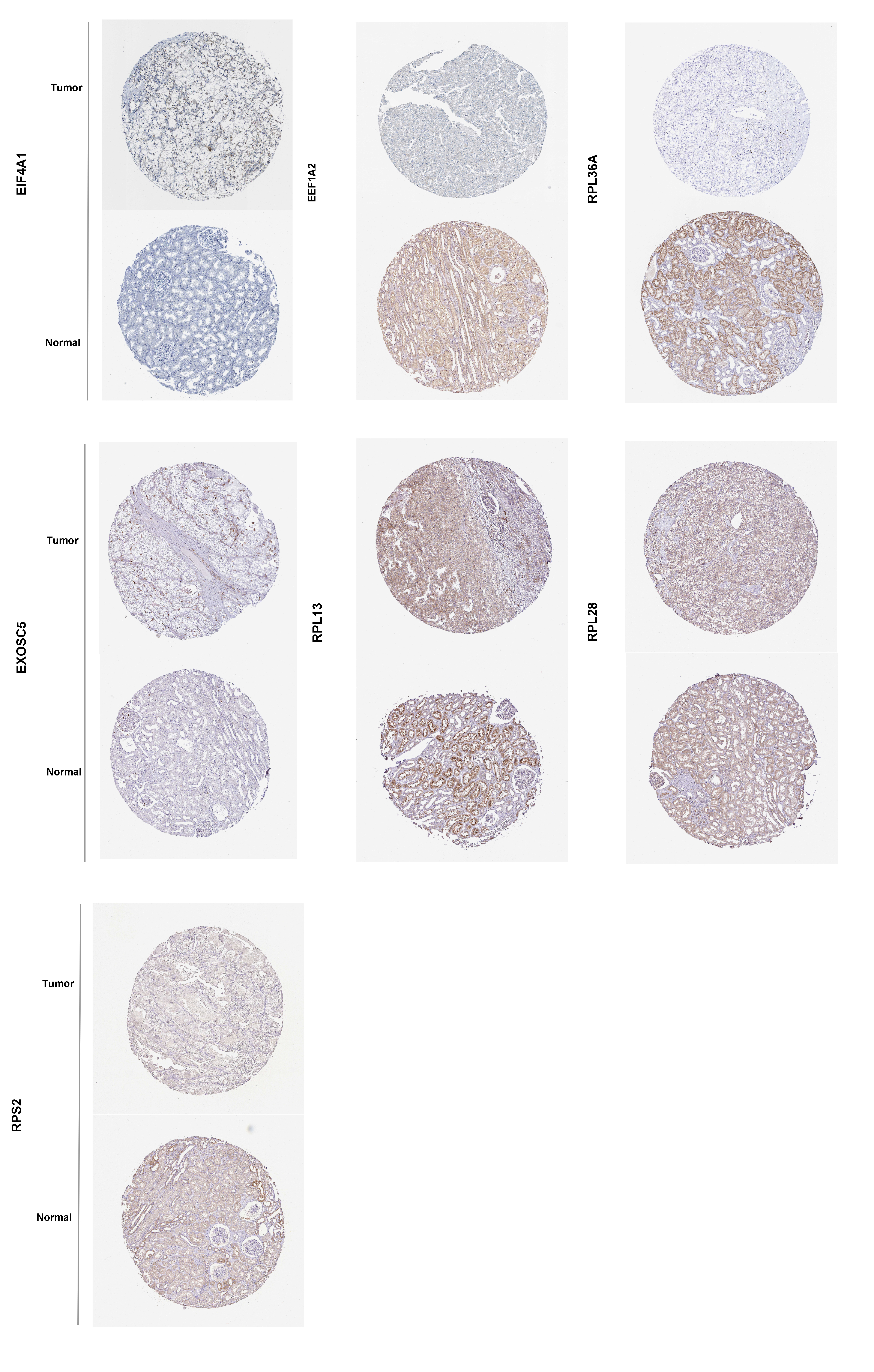

Supplement: Supplementary Figure 4 — Validation of the seven RBPs on the protein expression level based on Immunohistochemical results in KIRC; the data was retrieved from HPA database. [file Image_4.JPEG]
